# Supplementary material for: Are carbon emissions trading and green financial instruments synergistic? -Comprehensive quantitative research based on content analysis
Source: PLoS One. 2024 Mar 7;19(3):e0298601. doi: 10.1371/journal.pone.0298601 (PMC10919601; doi:10.1371/journal.pone.0298601)
Supplement: S1 Data — (ZIP) [file pone.0298601.s001.zip › Data/地级市Z_F工作报告.zip!/┴╔─■/╠·┴δ╩╨/└┤╫╘╣1⁄2╓┌║┼í╛┬φ┐╦╩2╛▌═°í┐_2006.docx]

各位代表：

现在，我代表市人民政府向大会报告工作，请予审议，并请市政协委员及其他列席会议的同志提出意见。

一、2005年工作回顾

过去的一年，我们在省委、省政府和市委的正确领导下，在市人大依法监督、市政协民主监督与大力支持下，团结和带领全市各族人民，坚持以邓小平理论和“三个代表”重要思想为指导，认真落实科学发展观，全面实施“五大战略”，聚精会神搞建设，一心一意谋发展，战胜了严重的洪涝灾害，成功地防控了禽流感疫情，使全市经济和社会发展呈现速度加快、效益提高、活力显现、后劲增强的好势头，国民经济和社会事业取得了显著成就，多项重点工作实现了历史性突破。

——国民经济增势强劲，速度和效益同步增长。全市GDP预计实现260亿元，增长18％，人均GDP达到1000美元。其中一二三产业增加值实现67亿元、113亿元和80亿元，分别增长12％、22．7％和15．4％。完成规模以上工业增加值80亿元，增长26％。完成全社会固定资产投资105亿元，增长32．9％。社会消费品零售额97亿元，增长13．6％。金融机构存款余额302.1亿元，贷款余额235．5亿元，分别比年初增长21．3％和14．6％。地区财政收入27亿元，增长25．2％；地方财政一般预算收入12．8亿元，增长26．4％；一般预算支出35．3亿元，增长27．9％。城镇居民人均可支配收入6300元，增长10．1％；农民人均纯收入3750元，增长7．7％。

——经济结构进一步优化，工业的主导地位初步显现。全市三次产业结构比例调整到25．7：43.5：30．8，工业增加值占GDP的比重达到36％。能源和优质农产品生产加工两大基地进一步确立，机械制造、化工医药、新型建材、食品加工等产业开始形成优势，高新技术产业形成一定规模；规模以上工业企业发展到550户，比上年增加200户；拥有省以上名牌产品18个。农业经济结构调整效果明显，粮食产量在大灾之年仍达到57．8亿斤，蔬菜等高效经济作物占种植业总面积的25％，畜牧业产值占农业总产值的比重达到50％；固定资产投资1000万元以上的农产品加工企业发展到40家，农业产业化总产值175亿元。第三产业有新的发展，结构层次有所提高。所有制结构日趋多元化，民营经济实现增加值130亿元，增长45％，占全市GDP比重达到50％。

——对外开放步伐加快，招商引资成效显著。共引进域外投资项目703个，到位资金104亿元。外商直接投资1254万美元，按新口径增长478％；外贸出口实现8347万美元，增长3％；外派劳务2640人，增长20％。投资超亿元的方向电子、沈阳机床集团西丰铸造、凯斯特电缆、嘉鑫有色金属、奥昌有色金属和人天科技等一批项目进展较快，有的已经投入生产。以打造沈铁工业走廊为重点的招商引资载体建设正在紧锣密鼓地进行，一些工业园区建设初见成效。

——基础设施建设力度加大，城乡面貌明显改观。投资6．1亿元，实施了集中连片棚户区改造工程，拆迁建筑面积82万平方米，建设回迁楼61万平方米，2000户居民在全省率先回迁新居。投资3．7亿元，完成了调兵山采煤沉陷区治理主体工程，总面积达到25万平方米。新建、改建市区主干道8条、街巷路24条、游园广场2个、公厕36座，完成了30条街巷路亮化工程，开工建设了垃圾处理厂。获得“中国优秀旅游城市”称号。加大城市环境综合整治力度，成功取缔了市区2768台客运人力三轮车和机动三轮车。小城镇建设迈出新步伐，修建道路918公里、排水沟142公里，新增自来水受益人口2．5万人。农村生产生活环境进一步改善。新建设黑色路面384．5公里，昌法线45公里二级路改造竣工通车，西丰县二级客运站建成并投入使用。整治河道险工险段26处，完成造林26．6万亩，治理水土流失面积20．5万亩，完成农村饮水工程83处，解决了6．2万人饮水困难问题。农机总动力达到162万千瓦，农业装备水平进一步提高。

——各项改革全面推进，市场体系逐步完善。国有企业改革取得突破性进展，87％的国有企业完成了产权制度改革。市直3户交通企业基本完成了民营化改制。市直和开原市、铁岭县、昌图县、西丰县粮食购销企业和商贸企业全部实现民营化。公用事业单位改革步伐加快，市煤气公司、液化气公司成功出售。积极推进农村税费改革，全面落实“两补一免”政策。西丰县山区和昌图县平原区集体林权制度改革试点工作进展顺利。商品交易市场不断扩大，劳务、技术、产权、人才、房地产等要素市场初步形成，城市经营性用地全部实行市场化运作，实现收入3．98亿元。审计、会计、法律服务等中介机构不断发展。

——科教兴市迈出较大步伐，社会事业全面发展。实施科技攻关及产业化项目60项，省级高新技术企业达到14家，实现高新技术产业产值28亿元。全年举办人才招聘会235场，达成协议2830人，引进国外专家96人次。加大了教育布局结构调整力度，撤并农村中小学93所，改造危房11万平方米，完成了农村中小学远程教育一期工程。职业教育招生规模不断扩大。铁岭师专与辽宁师范大学合作开办4个本科专业。完善了市县疾病预防控制体系和传染病防治体系，在铁岭县新型农村合作医疗试点基础上，昌图县、西丰县也正式启动。完成了市图书馆、少儿图书馆主体工程建设，举办了“中国·铁岭银冈书院学术研讨会”和“铁岭之夏”文化广场活动，秧歌戏《乡音乡情二人转》在中国第八届民间戏剧节上获奖。完成了64个村屯广播电视村村通工程，有线电视实现全市联网，广电大厦交付使用。体育工作再创佳绩，我市被省体育局授予“奥运贡献奖”。继续稳定低生育水平，人口自然增长率控制在3．3‰。深入开展了民族乡镇“手拉手，共同致富”帮扶活动。宗教事务管理得到强化。

——社会保障体系更加完善，人民生活质量有新的提高。实现就业再就业6．9万人，开发公益性岗位1650个，培训下岗失业人员2万人。大力实施“阳光工程”，新增劳务输出5．5万人。基本实现了养老、医疗、失业、工伤、生育“五险合一”，参保人数达124．9万人。积极巩固“两个确保”成果，有8．6万名企业离退休人员按时足额领取养老金，有1．3万名失业人员领到失业救济金；同时，有6．6万名城市低保对象领到最低生活保障金，实现了三条保障线的衔接。一个资金来源多样化、保障制度规范化、管理服务社会化的社会保障体系初步建立。农村低保人数达到7．9万人。开展大规模的扶贫帮困活动，筹集款物2100多万元，临时救助14．8万人，特困群众的基本生活得到保障。妥善安置受灾群众生产生活，为因洪灾倒塌房屋群众新建住房795户，维修住房1613户，对长期受洪灾威胁的两个村屯实施了整体搬迁。完成了22所中心敬老院建设。深入扎实地开展“平安铁岭”创建活动，被授予全省社会治安综合治理工作先进市。切实强化了安全生产工作，安全生产形势总体平稳。

——民主法制得到切实加强，精神文明建设取得丰硕成果。市政府自觉接受市人大的法律监督和市政协的民主监督，广泛听取各民主党派、工商联和无党派人士的意见和建议，定期向市人大常委会、市政协报告和通报工作。全年共办理人大代表建议105件，政协提案474件，办结率达到100％。加强了基层民主政治建设，进一步扩大了人民群众的民主权利。制定《铁岭市人民政府关于全面推进依法行政的实施意见》，政府法制工作取得进步。削减市级审批项目480多项。积极推进政务公开。建立健全教育、制度、监督并重的惩治和预防腐败体系，政府廉政建设得到加强。深入开展“双拥”活动，进一步密切了军政、军民关系。以经济共建、文化共建、环境共建为主要内容的城乡共建水平进一步提高，广泛开展了文明城市、文明村镇、文明行业、文明单位创建活动，全社会的思想道德素质进一步提高。

随着2005年各项任务目标的完成，“十五”计划也画上了一个圆满的句号。与2000年相比，全市GDP增长91．3％；地区财政收入增长113．2％，地方财政一般预算收入增长123．4％；规模以上工业增加值增长190％；全社会固定资产投资增长190％；社会消费品零售额增长68．9％；城镇居民人均可支配收入增长46．8％，农民人均纯收入增长49．8％。全市综合经济实力迈上一个新台阶，开始步入经济社会快速健康发展的新阶段。实践证明，开放立市、工业强市、城乡统筹、科教兴市、全民创业“五大战略”是正确的，是符合铁岭市情的。

各位代表，一年来及“十五”期间取得的各项成就，是省委、省政府和市委正确领导的结果，是市人大、市政协监督支持的结果，是全市人民同心同德、艰苦奋斗的结果。特别是各位人大代表、政协委员关心政府、理解政府、支持政府，以各种形式对政府工作提出许多极其宝贵的意见和建议，为我们不断改进工作提供了无穷的力量源泉。在此，我代表市人民政府，向辛勤工作在各条战线上的全市各族人民，向各位人大代表、政协委员，向各民主党派、工商联、无党派人士及人民团体，向中省直驻铁单位，向来铁岭投资的广大域外客商，向驻铁部队指战员、武警官兵，向所有关心支持铁岭改革开放和现代化建设的海内外朋友表示衷心的感谢并致以崇高的敬意！

在看到成绩的同时，我们也清醒地认识到，全市经济社会发展还存在不少长期积累、事关全局的深层次矛盾和问题。一是生产力还不发达，经济总量小，财政实力弱，城乡居民收入水平低。二是产业结构层次低，对外开放水平和城镇化水平低，增长方式还比较粗放。三是农民增收难度大，农业基础设施脆弱，农村各项社会事业发展滞后，“三农”问题仍很突出。四是深化改革任务艰巨，影响发展的体制性、机制性障碍还比较多。五是就业和社会保障压力大，全市城乡困难群众生活亟待改善，涉及群众切身利益的问题亟待解决，维护社会稳定任务繁重。六是政府职能转变滞后，一些部门工作效率低，服务意识较差，执政能力有待进一步提高。这些问题我们将高度重视，认真研究，并用发展和改革的办法逐步加以解决。二、“十一五”发展目标和主要任务“十一五”时期，是铁岭全面建设小康社会的关键时期。我们既面临着老工业基地振兴、沿海对外开放、加快辽西北地区开发以及沈阳经济区建设等重要机遇，同时也承受多重压力，任务十分艰巨。既要摆脱多年来形成的体制性、机制性、结构性矛盾的束缚，又要加快构建适应新形势、新任务的新体制和新机制；既要增加财政支出，解决好人民群众最关心、最直接、最现实的利益问题，又要千方百计筹措发展资金，把财政“蛋糕”做大；既要解决历史欠帐、走出现实困难，又要营造新优势、在激烈的竞争中赶超先进地区。在这样的背景和条件下，实现我市老工业基地振兴和构建和谐铁岭，就必须进一步解放思想，开拓创新，充分发挥后发优势，采取迎头赶上战略，实现经济社会超常规、跨越式发展。

“十一五”时期全市经济社会发展的指导思想是：以邓小平理论和“三个代表”重要思想为指导，全面贯彻落实党的十六届五中全会精神，以科学发展观统领经济社会发展全局，以老工业基地振兴和构建和谐铁岭为主题，以优化经济结构、转变经济增长方式为主线，以提升工业化、城镇化、产业化水平为重点，以打造沈铁工业走廊和各类工业园区为载体，以改革开放和自主创新为动力，以促进人的全面发展、不断提高人民群众物质文化生活水平为出发点和落脚点，坚定不移地实施“五大战略”，促进经济社会超常规、跨越式发展，努力把我市建成经济与社会、工业与农业、城市与农村、人与自然协调发展的充满生机与活力的新铁岭、大铁岭，为全面建设小康社会奠定坚实基础。

根据这一指导思想，我们把建设新铁岭、大铁岭作为“十一五”时期的总目标和政府工作的主旋律。“新铁岭”就是思想观念新、增长方式新、体制机制新、城乡面貌新、发展环境新、执政形象新；“大铁岭”就是经济总量大、城市规模大、结构调整步伐大、区域经济辐射力大、人民生活水平提高幅度大。发展的预期目标是：

——做大经济总量。到2010年，全市GDP达到650亿元，年均增长17％，力争实现780亿元，再造两个铁岭。地区财政收入实现80亿元，年均增长24．4％。实际利用外资和外贸出口实现重大突破。单位GDP综合能耗下降20％以上。

——社会事业全面进步。人口自然增长率控制在5‰左右。城镇登记失业率控制在5％以内。15岁以上人口平均受教育年限达到12年以上，体育人口达到40％。建立起比较完善的社会保障体系和城乡困难群众社会救助体系。

——城镇化迈上新台阶。城镇化率达到50％以上。将铁岭中心城市建成适宜居住、适宜创业、适宜休闲度假的生态山水名城。

——人民生活水平大幅度提高。城镇居民人均可支配收入达到1万元以上，年均增长11％。农民人均纯收入达到6000元以上，年均增长10％。

“十一五”期间全市经济社会发展的主要任务是：

1、优化经济结构，转变增长方式。加快经济结构战略性调整，不断优化第一产业，大力发展第二产业，努力繁荣第三产业。以主攻畜禽、发展蔬菜、扩大林果、优化粮豆为方向，进一步调整优化农业经济结构，促进农民增收和农村生产力发展。全力打造从新台子到毛家店南北一体，银州区——调兵山、开原——西丰东西两翼的沈铁工业走廊，使沈铁工业走廊成为全市产业的聚集带和经济的隆起带。着力建设能源和优质农产品生产加工“两大基地”。培育壮大先进制造、化工医药、新型建材、食品加工、高新技术“五大主导产业”。抓住一批投资亿元以上的大项目，扶大做强一批产值超亿元的骨干企业，培育一批省级以上名牌产品。到2010年，规模以上工业增加值占GDP的比重达到45％以上，形成以工业为主导的新型经济结构。改造提升传统第三产业，大力发展新兴第三产业，积极培育现代服务业。加快建设资源节约型、环境友好型社会，大力发展循环经济，切实转变经济增长方式。加强交通、水利、信息、电力等基础设施建设，提高发展的保障能力。

2、坚持城乡统筹，推进社会主义新农村建设。按照“生产发展、生活宽裕、乡风文明、村容整洁、管理民主”的要求和全面规划、典型引路、分步实施的原则，争取在“十一五”期间全市30％的村建成社会主义新农村。坚持以工业化为主导，城镇化为支撑，农业现代化为基础，大力发展县域经济，实现富民、强县、升位，使县域经济成为社会主义新农村建设的载体。以增加农民收入为目标，培植农村产业新优势；以农业产业化为牵动，大力发展新型农村合作经济组织；以基础设施为重点，加快建设新村镇；以城乡共建为依托，塑造农村新风貌；以提高农民素质为根本，积极培养有文化、懂技术、会经营的新型农民；以加快农村综合改革为动力，构建农村新体制。建立党委领导、政府负责、部门齐抓共管、全社会积极参与的工作机制，形成支持和参与社会主义新农村建设的合力。

3、加快推进城镇化进程，促进区域经济协调发展。按照“中心隆起、一体两翼、沿线组团、错位发展”的原则，以沈铁工业走廊为产业支撑，实现“城镇布局带状化，工业走廊一线牵”。做大做强中心城市。规划建设凡河新城区；使其成为适宜创业、适宜居住的行政服务中心、文化娱乐中心、休闲度假中心和新的居住中心；改造提升老城区，重点搞好柴河风光带建设，打造铁西商贸物流区，不断完善基础设施，增强中心城市的集聚力和辐射带动力。加快建设开原(清河)、昌图、调兵山、西丰四个副中心城市，使其成为辐射和拉动县域经济的支点。依托工业园区和交通优势，重点建设新台子、八面城、晓南等20个中心集镇，使其成为联结周边乡村、致富农民的结点。最终形成中心牵动、轴线辐射、城乡一体的城镇群、产业带、经济圈。

4、深化体制改革，扩大对外开放，提高自主创新能力。加快行政管理体制改革，做到政企分开、政事分开、政府与市场中介组织分开，基本形成行为规范、运转协调、公正透明、高效廉洁的行政管理体制。全面深化国有企业改革、农村各项改革、流通体制改革、投融资体制改革，分类推进事业单位改革，基本形成适应新形势、新任务的新体制新机制。坚定不移地实施开放立市战略，把招商引资与当地资源优势、产业优势、企业优势结合起来，切实提升招商引资质量，提高对财政税收的贡献率。“十一五”期间，全市要引进投资超亿元的大项目140个，总投资达到1000亿元。扩大出口规模，优化出口商品结构。加快建立以企业为主体、市场为导向、以攻关项目为载体、产学研相结合的技术创新体系，鼓励开发具有自主知识产权的高科技产品，切实增强引进消化吸收再创新、集成创新和原始创新能力。

5、坚持以人为本，加快构建和谐铁岭。实施积极的就业政策，以城镇困难群体、下岗失业人员和大中专毕业生为重点，抓好就业再就业工作，城镇登记失业率控制在5％以内。进一步完善社会保障体系，不断提高保障水平。优化配置教育资源，全面提高教育质量，促进各级各类教育协调发展，力争建成地方职业技术学院和本科大学。加强文化、体育基础设施建设，积极培育文化、体育产业。加快发展广播电视和新闻出版事业，形成广播、电视、网络、报纸四业并举，市县乡村四级联动，事业和产业全面协调持续发展的新格局。强化公共卫生体系、医疗服务体系、疾病预防控制体系建设，提高全市人民的健康素质和医疗服务水平，切实解决群众看病难看病贵问题。稳定人口低生育水平，积极推行优生优育，使人口增长与经济社会发展相协调。加强社区建设，完善服务功能，为和谐社会建设奠定坚实基础。协调好不同群体之间的利益关系，正确处理新形势下的人民内部矛盾。建立健全社会预警体系和应急救援机制，提高处置突发事件的能力。强化社会治安防控体系，建立安全生产长效管理机制，维护社会稳定。加大扶贫帮困力度，确保困难群体的基本生活。加强精神文明和民主法制建设，打造诚信铁岭。

三、2006年的主要工作

今年是实施“十一五”规划的开局之年，要确保经济社会又快又好发展，为建设新铁岭、大铁岭开好局、起好步。主要预期目标是：GDP实现320亿元，增长20％，单位GDP综合能耗下降3％。全社会固定资产投资200亿元，增长90％。地区财政收入32亿元，增长18．6％；地方财政一般预算收入15．3亿元，增长20％。社会消费品零售额115亿元，增长18％。城镇居民人均可支配收入7000元，增长11％，农民人均纯收入4050元，增长8％。

为实现上述目标，着力抓好以下九项重点工作：

(一)加快沈铁工业走廊建设，大力培育主导产业和骨干企业

集中力量建设沈铁工业走廊核心区。完成102线市区南出口到沈阳界双向八车道拓宽改造工程，确保7月底之前建成通车。搞好新台子高新技术园区2平方公里起步区的基础设施建设，达到“六通一平”，争取进入省级高新技术园区行列，引进资金20亿元。抓好懿路、腰堡、辽海工业园区基础设施建设，完成铁岭经济开发区扩区任务，争取引进一批大项目。加快开原铁西、调兵山开发区、昌图银河等县级工业园区建设。加强各级各类工业园区的统筹协调，形成合理的产业分工。围绕建设“两大基地”、“五大主导产业”，重点抓好铁岭电厂二期、亮中煤田开发、50万吨工业乙醇、4000吨新型干法水泥、10万吨电解铜、煤化工等50个投资亿元以上的重大项目，在土地征用、技术改造、银行贷款、财政贴息等方面予以大力支持。采取领导包扶、挂牌保护等措施，扶大做强“两电一煤”、陆平机器、铁新水泥、北绿集团等50户重点骨干企业。重点扶持球墨铸铁阀门、“北亚”药用植物油、“天淇”冷饮、“爱福尔”新型装饰材料等50个名牌产品。全市规模以上工业实现增加值104亿元，增长30％。

(二)扎实搞好“项目年”活动，务求对外开放和招商引资取得更大成效

以实施“523”工程为重点，认真组织开展“项目年”活动。要以大项目培育大企业，以大企业带动大产业，重点抓好工业项目、农业产业化项目、第三产业项目、基础设施项目和社会事业项目。一是明确任务，落实责任。继续实行领导包大项目责任制，实行全过程的跟踪服务，务求抓出实效。二是建立和完善科学考核体系。重点考核企业投入、上级专项等当年实际固定资产投资额，同时考核安置就业、上缴税金等效益指标。三是完善激励机制。建立奖励基金，实行以奖代补，对引进落地大项目的地区、部门和有功人员实行奖励。四是抓好项目源建设。建立市、县项目库，包装论证项目，满足项目开发的需要。通过“项目年”活动，确保开工建设投资500万元以上项目500个，投资总额突破200亿元，新增规模以上企业300户。五是切实加强外经外贸工作。实际利用外资3000万美元以上，实现翻一番。完成外贸出口1亿美元，增长30％以上。

(三)加快农村经济发展，确保社会主义新农村建设初见成效

把新农村建设与沈铁工业走廊建设和城乡共建有机结合起来，在铁南三镇等经济比较发达的乡镇和经济发展相对滞后的东部山区乡镇选择10％的村作为建设社会主义新农村的试点。一是大力发展县域经济。争取开原市、铁岭县进入省重点扶持的经济较发达县(市)行列，在抓好西丰县扶贫开发的同时，继续扶持昌图县加快发展。其它县(市)区也要实现在全省位次前移。二是加快东部山区开发。制定扶持东部山区发展的政策措施，充分利用和合理开发山区资源，大力发展苗木花卉、药材、林蛙、森林旅游等特色产业，促进农村经济协调发展。三是以农民增收为目标，继续调整优化农业结构。新建村外畜牧小区60个，实现畜牧业产值80亿元，占农业总产值的比重达到53％；新开发蔬菜10万亩，产量达到350万吨，增长20％；大力发展林业经济，林业产值增长25％以上；推进农业标准化，大力发展绿色食品。四是扩大农村劳务输出，做大劳务经济。继续实施“阳光工程”，搞好农民劳动技能培训，大力推广昌图五道村经验，提高劳务输出的组织化程度，转移农村剩余劳动力5万人。五是发展壮大龙头企业，加速推进农业产业化。新上固定资产投资2000万元以上的产业化龙头企业10家，重点规划建设25个种植业优势产区和6个畜牧业优势产区。大力发展新型农村合作经济组织，提高农民组织化程度，进一步解决分散的小生产与大市场的矛盾。六是改善农村生产生活设施。继续加强农村公路网建设，新增油路150公里。切实抓好“一池三改”工作，即建设沼气池、改厕、改圈、改厨，重点支持整村开发利用沼气。七是加快发展农村社会事业。探索农村“五站”建设的新路子，巩固和加强农村科技推广体系。继续推进“春晓工程”，加大对农村教育事业的投入，完善5所农村普通高中，新建和完善10所九年一贯制(寄宿制)学校，改造农村中小学危房6．3万平方米，完成农村中小学现代远程教育工程，实现校校通。加强农村公共卫生体系建设，深入开展爱国卫生运动，全面整治城乡环境。调整乡镇卫生院布局，集中力量办好20所中心卫生院和10所偏远地区卫生院，全面推开新型农村合作医疗。加强乡镇文化中心建设，继续推进广播电视“村村通”工程，丰富广大农民的文化生活。八是建立健全农村社会保障体系。积极推进农村养老保险制度，努力提高保障标准。九是加强农村的民主法制和精神文明建设。完善村民自治制度，不断推进农村民主政治建设。加强思想道德、科学文化建设，树立崇尚文明、崇尚科学的社会新风尚。

(四)大力发展第三产业，加快培育新的经济增长点

促进第三产业全面快速发展，充分发挥其在安置就业、繁荣市场、增加税收等方面的重要作用。完成城市商业网点规划修编，建设和培育南马路商业街，积极筹建银州区铁西、城南物流配送中心和开原靠山苗木花卉交易市场，建成铁岭果菜批发市场、废旧物资交易市场、西丰东北土特产品交易中心、开原五金城。继续实施“万村千乡”市场工程，加快农村新型合作连锁店建设，年内实现集中配送。严格实行粮食收购市场准入制度，维护粮食市场秩序，大力发展房地产、金融、保险、信息、法律服务等现代服务业，积极发展文化、体育、社区服务等需求潜力大的产业。做大旅游产业，实现旅游业总收入15亿元以上。

(五)规划建设凡河新城区，改造提升老城区功能，努力提高城镇化水平

全面完成城市总体规划和土地利用规划修编，结合铁岭县城搬迁，规划建设凡河新城区。按照百年一遇标准，搞好凡河治理；建设、恢复莲花湖湿地；抓好凡河、柴河向莲花湖调水工程；修建连接新老城区的交通主干道；结合社会主义新农村建设，做好新城区起步区内村屯的动迁、建设、安置工作；搞好环区绿化带、凡河两岸绿化带和莲花湖周边绿化带建设。改造提升老城区功能重点抓好12项工程：一是改造集中连片棚户区23万平方米；二是搞好市区绿化，新增公共绿地20公顷，行道植树3500株，争创省级园林城市；三是按照450平方米标准，建设26处社区办公用房；四是改造煤气中低压管线36公里，提高煤气供应质量；五是完成垃圾处理厂一期工程建设，确保6月底之前投入使用；六是改造城南污水排放系统，确保老城区污水排放不进入莲花湖；七是实施龙首山水土保持工程，对龙首山景点进行亮化；八是建设北市景观路；九是改造市区北出口；十是实施1．7万平方米的市客运中心改扩建工程；十一是实施市区公共交通增车加密工程；十二是筹建铁岭大剧院。继续加强城市管理，不断提升城市品位。加大经营城市力度，不断提高城市自我发展能力。在加强中心城市建设的同时，加大对县城、中心集镇和其他小城镇建设的扶持力度。加强与辽源市的配合协作，争取开工建设丰源铁路。

(六)积极推进各项改革，努力创新体制机制

一是全面完成国有工业企业产权制度改革任务，积极稳妥地推进非工业企业改革。支持各种所有制企业相互参股、收购和兼并国有企业，大力发展混合所有制经济。二是继续调整所有制结构，坚定不移地支持非公有制经济发展，民营经济增加值增长30％以上。三是全面深化农村改革。依法建立和完善农村土地流转制度和土地有偿使用制度，保护失地农民的合法权益。巩固农村税费改革成果，积极推进乡镇机构改革。基本完成集体林产权制度改革和动物卫生管理体制改革。四是加大公用事业单位改革力度，做到政企分开、事企分开。年内完成天信公司、焦化厂、污水处理厂改革任务。五是深化财政体制改革，进一步完善部门预算、国库集中收付、收支两条线和政府采购等制度，加强事业单位国有资产的监管，推行政府购买公共服务。六是积极稳妥地推进事业单位分类改革及其人事、分配制度改革。七是进一步抓好金融诚信体系建设和中小企业担保体系建设，加快城市信用社改革，年内组建商业银行。

(七)以人的全面发展为目标，加快发展各项社会事业

大力发展高新技术产业，实现高新技术产值36亿元。积极申报市213研究所为“颅面鉴定国家重点试验室”。完成高新技术产业园区孵化器建设，入孵企业10家。抓好种子工程，优良品种覆盖率达到95％以上。继续抓好人才引进与培养，努力开创人才工作的新局面。进一步加大对教育事业的投入，建设好城市标准化初中，完成铁岭高中等3个重点基建项目。努力扩大职业院校办学规模，整合职业教育资源，筹建地方高等职业技术学院。加快文化设施建设，加强文化、出版、文物市场管理，制定扶持政策，促进文化产业加快发展。举办第二届小品小戏二人转大赛及首届城乡广场活动大赛，丰富群众文化生活。加强卫生基础设施建设，进一步改善医疗卫生条件，提高人民群众的医疗卫生保障水平。继续稳定低生育水平，加大出生人口性别比升高问题综合治理力度。巩固广播电视村村通成果，构建广播电视节目摄录播出数字化、传输网络化新体系。构建全民健身服务体系，增加体育人口，培养体育人才，力争在省十运会上取得好成绩。加强城乡精神文明建设，争取进入全省文明城市创建工作先进市。加大双拥共建工作力度，争创全国“双拥模范城”。认真落实党的宗教政策，依法加强宗教事务管理，引导宗教界和广大信教群众为经济社会发展作贡献。

(八)建立健全公共财政体系，切实关心群众生产生活

进一步调整优化财政支出结构，确保科技、教育、农业等法定支出稳步增长。大力开展增收节支活动，招待费、出国经费、会议费在上年基础上压缩30％，公用经费压缩20％，节省的资金全部用于救助困难群众。加强以“五险合一”为重点的社会保障体系建设，继续扩大城乡低保覆盖面，逐步提高保障标准。建立健全激励机制和服务体系，激发全民创业热情，做到普通百姓创家业，能人精英创企业，领导干部创事业，鼓励下岗职工和大学毕业生自主创业，鼓励事业单位人员转岗创业，通过全民创业促进就业和再就业，全年净增就业岗位5万个，确保零就业家庭至少有一人就业，城镇登记失业率控制在5％以内。继续抓好农村扶贫开发工程，加大整村脱贫工作力度。进一步完善城乡困难群体社会救助体系。搞好临时救助救济，帮助困难家庭解决子女上学、医疗救治、住房、取暖等实际问题。为农村特困户新建住房150户，维修住房400户，做好优抚、救灾、救济工作，保障特困群众的基本生活。加快发展社会福利事业，筹建妇女儿童救助保护中心。加强公用事业建设，保证居民的供热、供气、供水、供电。严厉打击制售假冒伪劣商品行为，为人民群众创造安全放心的购物环境。切实加强信访工作，确保群众反映的合理诉求在最短时间内得到解决。进一步完善社会治安防控体系，严厉打击、积极预防各种犯罪，增强人民群众的安居感。认真开展“五五”普法工作，加大基层排查和化解矛盾纠纷的力度，预防和妥善处置各种突发事件，确保社会稳定。加强安全生产监管工作，认真整治事故隐患，坚决避免重特大安全事故的发生。

(九)加快转变政府职能，积极推进依法行政

切实加强政府自身建设，为全面完成各项任务提供有力的组织保障。一是加快转变政府职能。更加注重履行社会管理和公共服务职能，把政府管理经济的职能切实转变到主要为市场主体服务和创造良好发展环境上来。继续深化行政审批制度改革，市级行政审批项目由139项削减到92项，成为全国保留最少的城市之一；向县(市)区下放市级行政管理权31项，实现管理重心下移；将大部分行政执法权移交给县(市)区行政主管部门，解决多头执法、重复执法、交叉执法问题。加强各级公共行政服务中心建设，提高办事效率，努力建设服务型政府。二是积极推进依法行政。全面落实《铁岭市人民政府关于全面推进依法行政的实施意见》，严格按照法定权限和程序行使权力，履行政府职责。自觉接受人大及其常委会的依法监督和人民政协的民主监督，认真执行人大及其常委会的各项决议，加强和改进人大代表建议、政协提案办理工作，提高依法行政的能力和水平，努力建设法制政府。三是大力推进政务公开，进一步拓宽与民主党派、人民团体及社会各界人士的沟通和联系渠道，保障人民群众的利益诉求权利和民主权利，努力建设公正透明政府。四是以实施《公务员法》为契机，全面加强公务员队伍建设。抓好学习型机关建设，大力倡导认真学习的风气、研究问题的风气、积极探索的风气，用科学发展观武装各级领导干部的头脑，不断提高政府的行政效能和服务质量，努力建设高效政府。五是切实转变工作作风，真抓实干。树立正确的政绩观，塌下心来想问题，扑下身子干实事，深入基层调查研究，抓紧时间解决问题，集中力量攻坚克难，确保各项任务落到实处，努力建设务实政府。六是加强廉政勤政建设。继续抓好领导干部廉洁自律工作，时刻牢记“两个务必”，坚持权为民所用、情为民所系、利为民所谋，树立领导干部的良好形象。大力纠正部门和行业不正之风，从源头上预防和治理腐败现象和不廉洁行为，努力建设廉政为民政府。

各位代表，五年的蓝图已经绘就，今年的任务已经明确，让我们在市委的领导下，以奋发有为的精神状态和求真务实的工作作风，团结奋进，开拓创新，为建设新铁岭、大铁岭，为300万铁岭人民过上幸福、富裕、文明的新生活而努力奋斗！
